# Comparison of Different Glycosidase Activities in Conditions of Cancer

**DOI:** 10.1038/bjc.1957.59

**Published:** 1957-09

**Authors:** J. Conchie, G. A. Levvy


### COMPARISON OF DIFFERENT GLYCOSIDASE ACTIVITIES IN CONDITIONS OF CANCER

J. CONCHIE AND G. A. LEVVY
*From the Rowett Research Institute, Bucksburn, Aberdeenshire*

Received for publication June 4, 1957

CONSIDERABLE attention has been paid to the enzyme $\beta$-glucuronidase in connection with cancer. Physiological aspects of the action of this enzyme have recently been reviewed by Levvy (1956). Various workers have found that the $\beta$-glucuronidase activity of malignant tumours in both humans and mice is high in comparison with that of most normal organs, and usually much higher than that of the surrounding healthy tissue. It has also been suggested (Odell and Burt, 1950) that abnormally high figures for $\beta$-glucuronidase activity in human vaginal fluid have diagnostic significance in uterine cervical cancer, particularly prior to the menopause.

$\beta$-Glucuronidase is practically ubiquitous in its distribution in the animal body, but its activity in a given tissue bears no relation to the general metabolic activity nor the oxygen uptake of the tissue. One possible function of the enzyme is the hydrolysis of steroid glucuronides to release the active hormones, but this provides no satisfactory explanation of the response in enhanced or diminished activity of the enzyme in different organs to the administration of steroid hormones, a phenomenon that has been extensively studied. It has been shown (Linker, Meyer and Weissmann, 1955) that $\beta$-glucuronidase and hydrolyses degradation products from chondroitin and hyaluronic acid, and it may well be that a rôle in mucoid catabolism is at least as important a function of $\beta$-glucuronidase in the animal body. Such a rôle might be expected to assume major proportions in tissue growth, repair or proliferation, as well as after steroid hormone administration, all these being conditions under which an enhancement of $\beta$-glucuronidase activity has been frequently observed.

On this line of reasoning, it was considered that $\beta$-glucuronidase might be in no way unique amongst glycosidases in its behaviour *in vivo*, and it has in fact recently been found (Conchie, Findlay and Levvy, 1956, 1957) that $\beta$-*N*-acetylglucosaminidase and the newly-discovered $\alpha$-mannosidase in mammalian tissues do respond to steroid hormone administration. $\beta$-*N*-Acetylglucosaminidase has also been shown to participate in the catabolism of hyaluronic acid (Linker *et al.*, 1955), whilst it has been tentatively suggested that $\alpha$-mannosidase may have a similar role in the metabolism of mucoids containing mannose (Conchie *et al.*, 1956, 1957; Conchie and Mann, 1957). The present communication deals with these enzymes in conditions of cancer, in comparison with $\beta$-glucuronidase.

### MATERIALS AND METHODS

### *Materials*

*p*-Nitrophenyl $\alpha$-D-mannoside was prepared as described by Jermyn (1955) and had m.p. 176° and $[\alpha]^{19}_D + 145°$ (c, 0·2 in water).

Phenyl *N*-acetyl $\beta$-D-glucosaminide was prepared as described by Conchie and Levvy (1957).

Phenolphthalein $\beta$-D-glucuronide was prepared biosynthetically (Talalay, Fishman and Huggins, 1946).

The ascitic tumours S37, Ehrlich and 2146 were obtained from Professor J. S. Young, Department of Pathology, University of Aberdeen, and were transplanted weekly into our laboratory strain of white mice (Tyler's Original: Mill Hill colony).

A frozen suspension of Tumour 2146 was obtained from Dr. J. Craigie, Imperial Cancer Research Fund, and injected into our laboratory mice.

Mice with spontaneous mammary tumours, and those implanted with Crocker sarcoma 180, were obtained from Professor A. Haddow, Chester Beatty Research Institute.

Vaginal fluid samples were collected by Dr. J. G. Lawson, Department of Midwifery, University of Aberdeen, who also supplied the specimens of genital tumours.

Samples of normal human kidney tissue and of Grawitz tumour were supplied by Dr. A. J. Carr, Department of Pathology, University of Aberdeen.

### *Methods*

*Enzyme preparations.*—All tissues to be assayed were first homogenised in water with a glass homogeniser. The suspensions were diluted so that under the conditions of assay they liberated from the substrate suitable amounts of aglycone (80–100 $\mu$g. *p*-nitrophenol, 40–50 $\mu$g. phenol and 30–40 $\mu$g. phenolphthalein). In the case of $\beta$-*N*-acetylglucosaminidase assays, the homogenates, after incubation for 1 hour at 37° with 0·05 M citric acid buffer pH 4·3, followed by the addition of 0·15 M sodium chloride (Pugh, Leaback and Walker, 1957*a*), were centrifuged and the supernatant fractionated with saturated ammonium sulphate, the fraction precipitated between 20 and 80 per cent saturation being used for assay. This avoided the high enzyme blanks usually obtained with crude homogenates when estimating phenol.

Ascitic fluids were either diluted with water and homogenised or were mixed with Triton X-100 (Rohm & Haas Company—0·6 per cent final concentration) to rupture the cells before assay.

Vaginal fluid samples were also diluted and homogenised. In view of the small amounts of fluid available, incubations were carried out for 2 hours instead of 1 hour in the case of $\alpha$-mannosidase assays.

*Assays.*—Estimations of $\alpha$-mannosidase activity were carried out in phosphate-citrate buffer pH 4·6 with *p*-nitrophenyl $\alpha$-mannoside as substrate. To 3 ml. of buffer were added 0·5 ml. of 0·016 M substrate and 0·5 ml. enzyme solution and the mixture incubated for 1 hour at 37°. The reaction was stopped by the addition of 2 ml. trichloroacetic acid (5 per cent w/v), and 4 ml. of the reaction mixture neutralised with NaOH and developed with glycine-$Na_2CO_3$ buffer as described by Conchie (1954) for $\beta$-glucosidase assays. The free *p*-nitrophenol was measured on the Spekker photoelectric absorptiometer using an Ilford 601 violet filter.

$\beta$-*N*-Acetylglucosaminidase activity was measured by the method of Kerr, Graham and Levvy (1948), the liberated phenol being measured using an Ilford

No. 608 red filter in the absorptiometer. The conditions of assay were those described by Pugh, Leaback and Walker (1957*b*).

*β*-Glucuronidase activity was measured by the method described by Levvy (1952) for mouse-liver *β*-glucuronidase.

### RESULTS

Table I shows mean figures for the *α*-mannosidase, *β*-glucuronidase and *β*-*N*-acetylglucosaminidase activities of various mouse tumours, in comparison with figures for uninvolved liver in the same groups of mice. Liver is among the richer tissues in all three enzyme activities. With the exception of the C3H mice, the animals were drawn from various mixed breeding colonies. Spontaneous mammary tumours and Crocker sarcoma 180 showed activity levels for all three enzymes of the same order as those seen in liver. It should be noted that the C3H strain is one with a genetically-determined low *β*-glucuronidase activity in all tissues (Law, Morrow and Greenspan, 1952). It is also, in our experience, a very unthrifty and infertile strain. Cohen and Bittner (1951) showed that the hereditary factor extended to *β*-glucuronidase in cancer tissue. It is evident from our results that the *α*-mannosidase and *β*-*N*-acetylglucosaminidase activities are not genetically linked to *β*-glucuronidase activity.

TABLE I.—*Enzyme Activities in Mouse Tumours*

For conditions of assay see text. Results are expressed in μg. *p*-nitrophenol, phenolphthalein or phenol liberated per g. of tissue (wet weight) in 1 hour. Uninvolved liver shown for comparison.

Values are given as mean ± standard error followed (in parentheses) by the number of animals in the group.

| | Enzyme activities | | |
|---|---|---|---|
| Tumour | α-Mannosidase | β-Glucuronidase | β-*N*-Acetyl-glucosaminidase |
| Spontaneous mammary tumour : | | | |
| Mixed strain : Tumour | 3,750±440 (9) | 2,280±130 (9) | 7,830±1,350 (5) |
| Liver | 1,352 | 2,631 | 7,312 |
| C3H strain : Tumour | 1,320±4 (2) | 650±48 (2) | 3,937±120 (2) |
| Liver | 1,311 | 319 | 6,170 |
| Crocker sarcoma 180 (subcutaneous injection) : | | | |
| Tumour | 1,150±85 (9) | 2,100±680 (5) | 4,120±500 (7) |
| Liver | 2,587 | 2,498 | 9,030 |
| Ascitic tumours (intraperitoneal injection):* | | | |
| S37 tumour | 112±4 (3) | 140±16 (2) | 1,190±90 (2) |
| Ehrlich tumour | 114±11 (3) | 207±40 (3) | 1,400±130 (2) |
| 2146 tumour | 116±14 (3) | 146±3 (2) | 1,240±200 (2) |
| Liver | 1,572 | 2,300 | 10,430 |
| Subcutaneous injection of ascitic fluid : | | | |
| 2146 tumour (frozen)† | 600±60 (5) | 2,060±42 (5) | 5,890±300 (3) |
| 2146 tumour (fresh)† | 480±50 (13) | 1,880±200 (13) | 7,570±700 (13) |
| 2146 tumour (fresh : 2nd subcutaneous transplant) | 490±37 (9) | 1,540±110 (9) | 7,470±710 (4) |
| Ehrlich tumour | 720±70 (2) | 1,940±390 (2) | 7,430±560 (2) |
| Liver | 1,604 | 2,927 | 7,781 |

* Results expressed per ml. ascitic fluid.

† These tumours were of diverse origin, the second specimen have been kept in the ascitic form for several generations.

Ascitic tumours would appear to be a major exception to the generalisation that tumours have a relatively high β-glucuronidase activity, since they had only about one-sixteenth the value for liver. This difference between ascitic and other tumours extended to α-mannosidase and β-*N*-acetylglucosaminidase activity.

Thus far it can be seen that both the α-mannosidase and β-*N*-acetylglucosaminidase activities of tumours paralleled their β-glucuronidase activity. After subcutaneous injection of tumours that had been through the ascitic phase, however, α-mannosidase displayed a low figure in the tumour in comparison with that for liver, whereas β-glucuronidase and β-*N*-acetylglucosaminidase displayed similar values to those observed with spontaneous mammary tumours and Crocker sarcoma 180. As regards the latter two enzymes at least, the site of injection would appear to be the sole determining factor for the enzyme activity of the resultant tumour.

A few figures for human surgical specimens are shown in Table II. From these few results it cannot be said that the β-glucuronidase activity of cancerous tissues

TABLE II.—*Enzyme Activities in Some Human Tumours and in Uninvolved Tissue*

For conditions of assay see text. Results are expressed in μg. *p*-nitrophenol, phenolphthalein or phenol liberated by 1 g. tissue (wet weight) in 1 hour.

| | | | Enzyme activities | | |
|---|---|---|---|---|---|
| Subject | Age | Specimen | α-Mannosidase | β-Glucuronidase | β-*N*-Acetylglucosaminidase |
| Mrs. J. C— | 65 | Cancerous vulva | 715 | 446 | 7,943 |
| Mrs. C. W— | 41 | Malignant anterior lip of cervix | 721 | 479 | 7,021 |
| | | Non-malignant posterior lip of cervix | 218 | 479 | 6,211 |
| Mrs. H. O— | 47 | Cancerous uterine body | 818 | 818 | 19,900 |
| Mrs. M. McK— | 64 | Grawitz tumour | 351 | 635 | 4,301 |
| | | Normal kidney tissue | 1,105 | 1,793 | 22,040 |

was any higher than that of the corresponding healthy tissue, nor did this appear to be true for α-mannosidase and β-*N*-acetylglucosaminidase. It can, however, be seen that α-mannosidase and β-*N*-acetylglucosaminidase activities in human tissues are at least comparable with that of β-glucuronidase.

Table III gives individual figures for the activities of the enzymes in vaginal fluid from human subjects, with and without genital cancer. Figures for β-glucuronidase were obtained by Dr. J. G. Lawson. It has been stated that a vaginal fluid β-glucuronidase activity of 400 units per g. fluid distinguishes normal premenopausal women from those with cancer of the uterine cervix, 80 to 90 per cent of the women falling into the correct category (Kasdon, Homburger, Yorshis and Fishman, 1953): healthy postmenopausal women frequently display a high value, the enzyme in vaginal fluid being apparently under ovarian control. The data in Table III suggest that a similar test might be founded on the α-mannosidase activity of vaginal fluid, with a dividing line at about 200 units per g. fluid, and with the added advantage that in healthy women zero values are frequently observed for this enzyme : the only false high value was in a post-menopausal woman. It is interesting to recall in this

TABLE III.—*Enzyme Activities in Vaginal Fluids*

For conditions of assay see text. Results are expessed as $\mu$g. of *p*-nitrophenol, phenolphthalein or phenol liberated per g. of fluid in 1 hour.

(*a*) Vaginal fluids from cancer patients

| Subject | Age | Menopause | Condition | Enzyme activities: α-Mannosidase | β-Glucuronidase | β-*N*-Acetylglucosaminidase |
|---|---|---|---|---|---|---|
| Mrs. T. B— | 48 | Pre | Carcinoma of cervix (untreated) | 246 | 566 | — |
| Mrs. C. C— | 76 | Post | Advanced carcinoma of cervix and vagina (2 days after radium treatment) | 1350 | 1266 | — |
| | | | Four days after radium treatment | 540 | 335 | — |
| Mrs. A. M— | 61 | Post | Carcinoma of cervix (2 days after radium treatment) | 155 | 186 | — |
| Mrs. C. W— | 41 | Pre | Carcinoma of cervix (untreated) | *Nil* | 405 | — |
| | | | Three weeks after first radium treatment | 652 | 768 | 2015 |
| Mrs. M. T— | 70 | Post | Carcinoma of cervix (untreated) | 923 | 747 | 2418 |
| Mrs. H. O— | 47 | Pre | Cancer of uterine body with spread down vagina (untreated) | 413 | 337 | 1399 |
| Mrs. J. C— | 65 | Post | Advanced carcinoma of vulva (untreated) | *Nil* | 230 | — |

(*b*) Vaginal fluids from non-cancer subjects

| Subject | Age | Menopause | Enzyme activities: α-Mannosidase | β-Glucuronidase | β-*N*-Acetylglucosaminidase |
|---|---|---|---|---|---|
| Mrs. J. L— | 32 | Pre | *Nil* | 100 | — |
| Mrs. E. H— | 37 | Pre | 171 | — | 2568 |
| Mrs. I. T— | 46 | Pre | *Nil* | — | 108 |
| Mrs. I. D— | 51 | Post | *Nil* | — | 491 |
| Mrs. I. B— | 41 | Pre | 174 | — | 814 |
| Mrs. I. S— | 70 | Post | 1064 | — | 3483 |
| Mrs. C. R— | 44 | Pre | 321† | — | 5142† |
| Miss I. R— | 24 | Pre | — | — | 347 |
| Mrs. E. D— | 26 | Pre | — | — | 811 |

† Non-malignant fibroid growth in uterus.

connection that ovariectomy reduces mouse uterine $\alpha$-mannosidase activity to vanishingly small proportions (Conchie, Findlay and Levvy, 1957), whereas the $\beta$-glucuronidase activity is only reduced to about half by this measure (Kerr, Campbell and Levvy, 1949; Fishman and Fishman, 1944; Fishman and Farmelant, 1953). $\beta$-*N*-Acetylglucosaminidase activity did not appear to distinguish between normal women and cancer cases, but the results for this enzyme are too few to form any definite opinion.

### DISCUSSION

$\beta$-Glucuronidase can no longer be regarded as unique amongst mammalian glycosidases, since $\alpha$-mannosidase and $\beta$-*N*-acetylglucosaminidase show an equally wide distribution in the body, respond to steroid hormone administration and, as we have just seen, show similar activities in cancer tissue as compared with appropriate healthy tissue values. It would also appear that the correlation between high $\beta$-glucuronidase activity and malignant growths is not as invariable as has been generally believed. The relatively very low $\beta$-glucuronidase activity of ascitic tumours is particularly perplexing, unless the high $\beta$-glucuronidase activity of most other tumours is associated with the invasion of solid body tissues. With regard to $\beta$-glucuronidase assay as an aid in the diagnosis of uterine cervical cancer, it would appear that $\alpha$-mannosidase assay might be equally well employed, perhaps with advantage.

The secretion of $\beta$-*N*-acetylglucosaminidase in normal vaginal fluid is of particular interest in view of the very high activity of this enzyme in the semen of all species, including man (Conchie and Mann, 1957).

There is no evidence whatsoever for the conjugation of steroid hormones or their metabolites with mannose or *N*-acetylglucosamine, and there can thus be no attempt to explain an enhanced activity of either $\alpha$-mannosidase or $\beta$-*N*-acetylglucosaminidase in terms of an adaptation by the organism to meet a need for increased metabolism of these hormones. It is much more probable that a high activity of these enzymes, whether caused by steroid hormones or other means, reflects an increase in mucoid catabolism, and this same explanation may very well extend to $\beta$-glucuronidase. The fact that steroid hormones, as well as altering $\beta$-glucuronidase activity *in vivo*, form conjugates that are hydrolysed by this enzyme can thus be regarded as purely coincidental.

Maintaining an overall balance in the body with the forces of synthesis would appear to provide a vital function for hydrolytic enzymes such as these, quite apart from any specialised action, such as the release of steroid hormones from conjugates by $\beta$-glucuronidase.

### SUMMARY

Mouse and human malignant tumours as well as vaginal fluid from cases of uterine cervical cancer, have been examined for their $\alpha$-mannosidase and $\beta$-*N*-acetylglucosaminidase activity. In most respects these two enzymes resembled $\beta$-glucuronidase, an enzyme that has been extensively studied in connection with cancer. Ascitic tumours displayed astonishingly low values for $\beta$-glucuronidase activity as well as for the other two enzymes examined. The preliminary results suggested that $\alpha$-mannosidase assay in vaginal fluid might be as useful as a diagnostic aid in uterine cervical cancer as the measurement of $\beta$-glucuronidase activity.

We are indebted to Mr. A. J. Hay for competent technical assistance.

### REFERENCES

Cohen, S. L. and Bittner, J. J.—(1951) *Cancer Res.*, **11**, 723.
Conchie, J.—(1954) *Biochem. J.*, **58**, 552.
*Idem*, Findlay, J. and Levvy, G. A.—(1956) *Nature*, **178**, 1469.—(1957) *Biochem. J.*, **65**, 18P.
*Idem* and Levvy, G. A.—(1957) *Ibid.*, **65**, 389.
*Idem* and Mann, T.—(1957) *Nature*, **179**, 1190.
Fishman, W. H. and Farmelant, M. H.—(1953) *Endocrinology*, **52**, 536.
*Idem* and Fishman, L. W.—(1944) *J. biol. Chem.*, **152**, 487.
Jermyn, M. A.—(1955) *Aust. J. Chem.*, **8**, 403.

KASDON, S. C., HOMBURGER, F. YORSHIS, E. AND FISHMAN, W. H.—(1953) *Surg. Gynec. Obstet.*, **97**, 579.
KERR, L. M. H., CAMPBELL, J. G. AND LEVVY, G. A. —(1949) *Biochem. J.*, **44**, 487.
*Idem,* GRAHAM, A. F. AND LEVVY, G. A.—(1948) *Ibid.*, **42**, 191.
LAW, L. W., MORROW, A. G. AND GREENSPAN, E. M.—(1952) *J. nat. Cancer Inst.*, **12**, 909.
LEVVY, G. A.—(1952) *Biochem. J.*, **52**, 464.—(1956) *Vitam. & Horm.*, **14**, 267.
LINKER, A., MEYER, K. AND WEISSMANN, B.—(1955) *J. biol. Chem.*, **213**, 237.
ODELL, L. D. AND BURT, J. C.—(1950) *J. Amer. med. Ass.*, **142**, 226.
PUGH, D., LEABACK, D. H. AND WALKER, P. G.—(1957*a*) *Biochem. J.*, **65**, 16P.—(1957*b*) *Ibid.*, **65**, 464.
TALALAY, P., FISHMAN, W. H. AND HUGGINS, C.—(1946) *J. biol. Chem.*, **166**, 757.


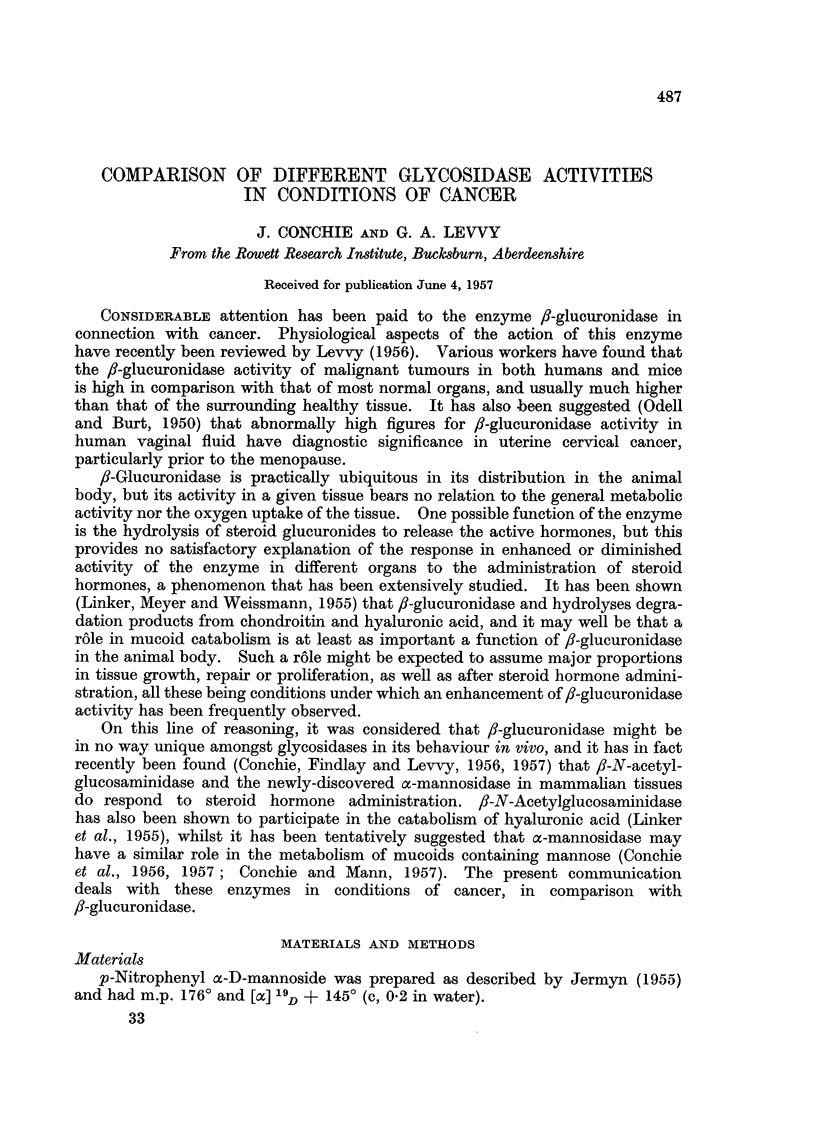

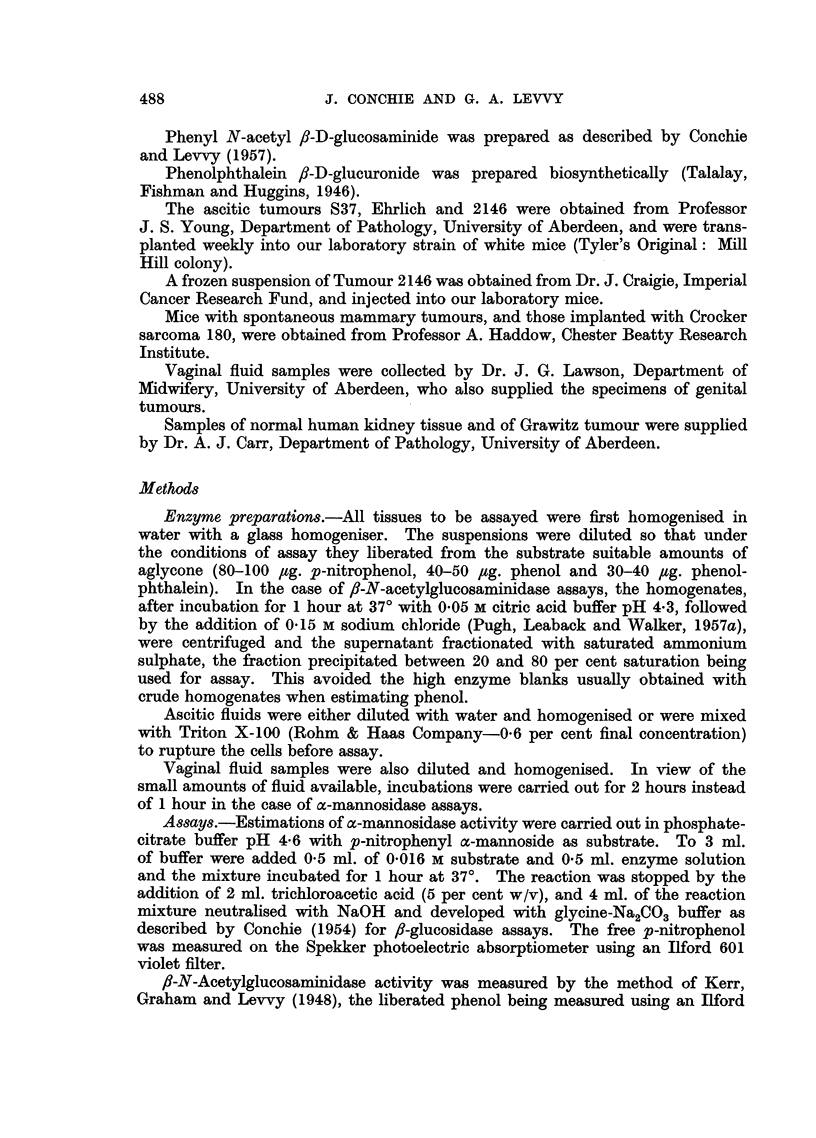

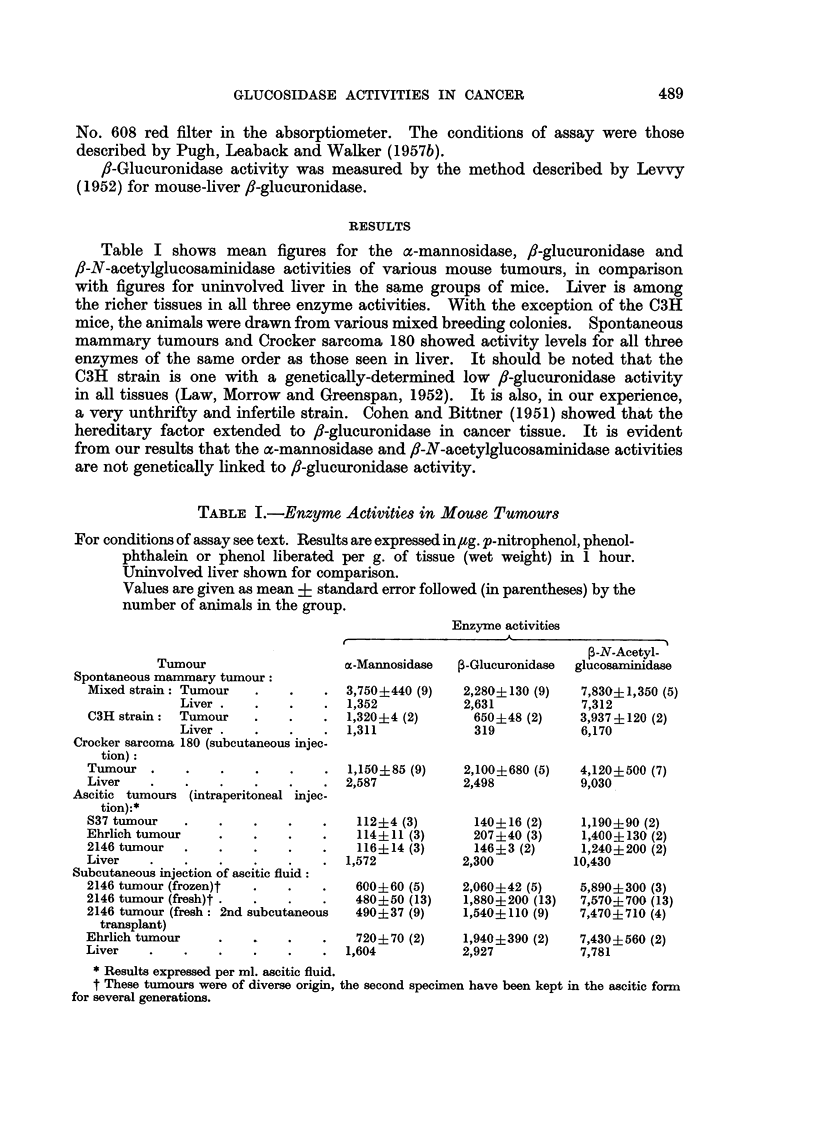

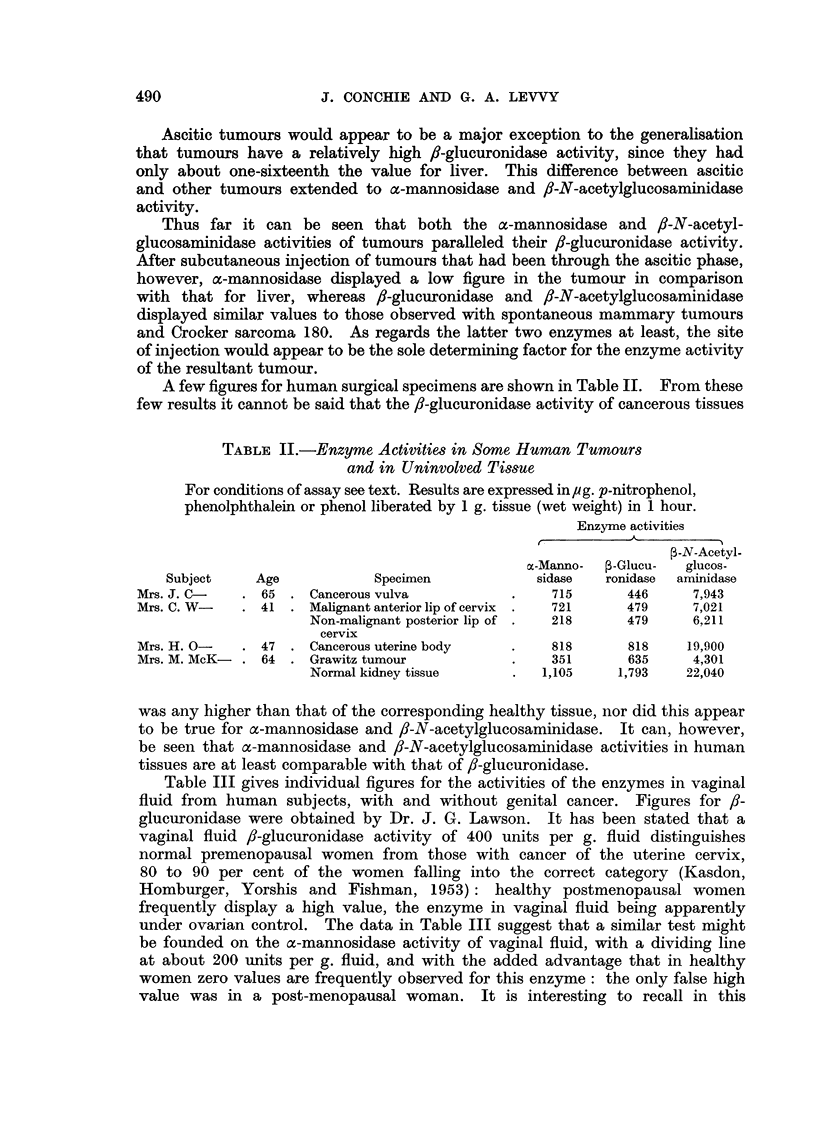

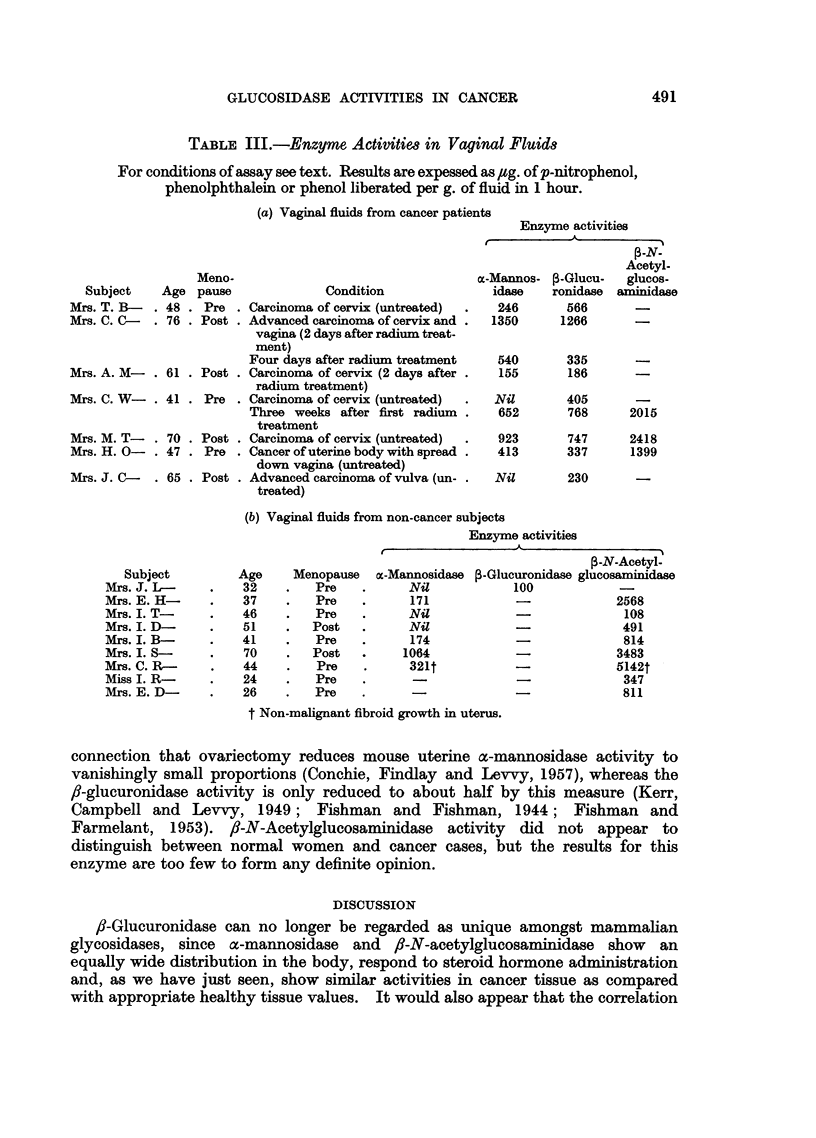

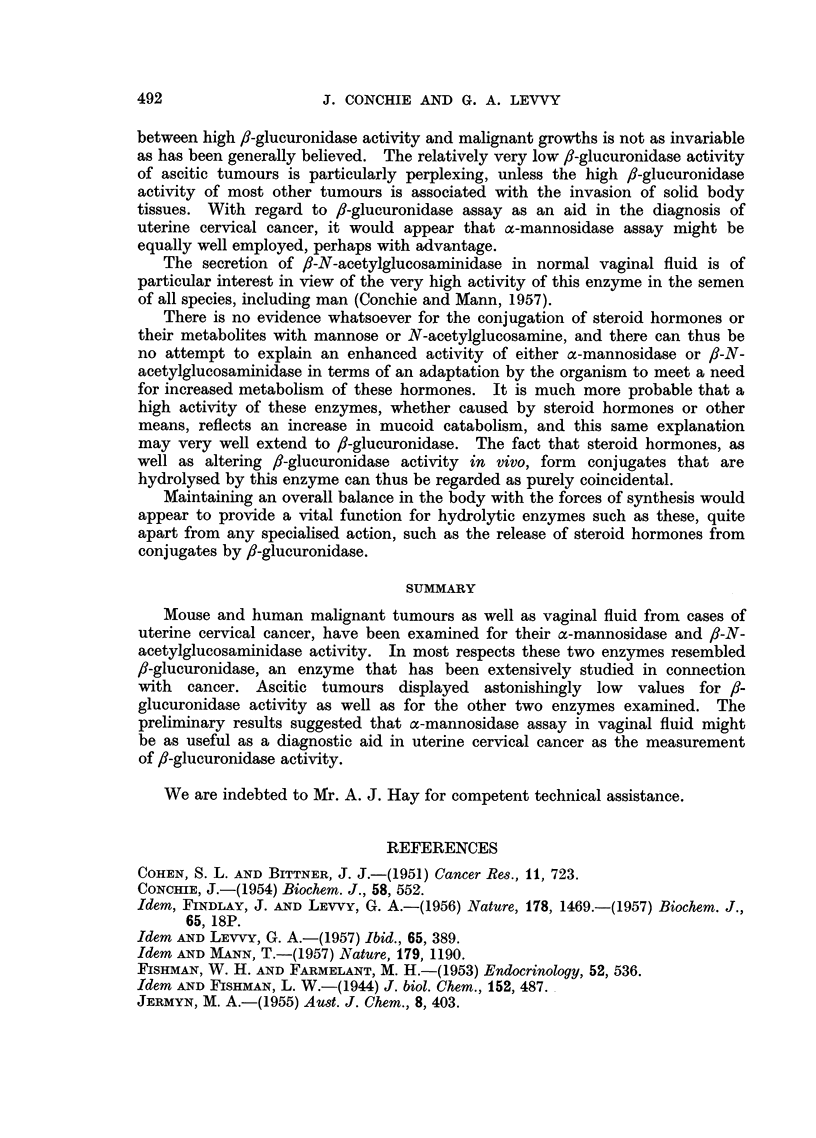

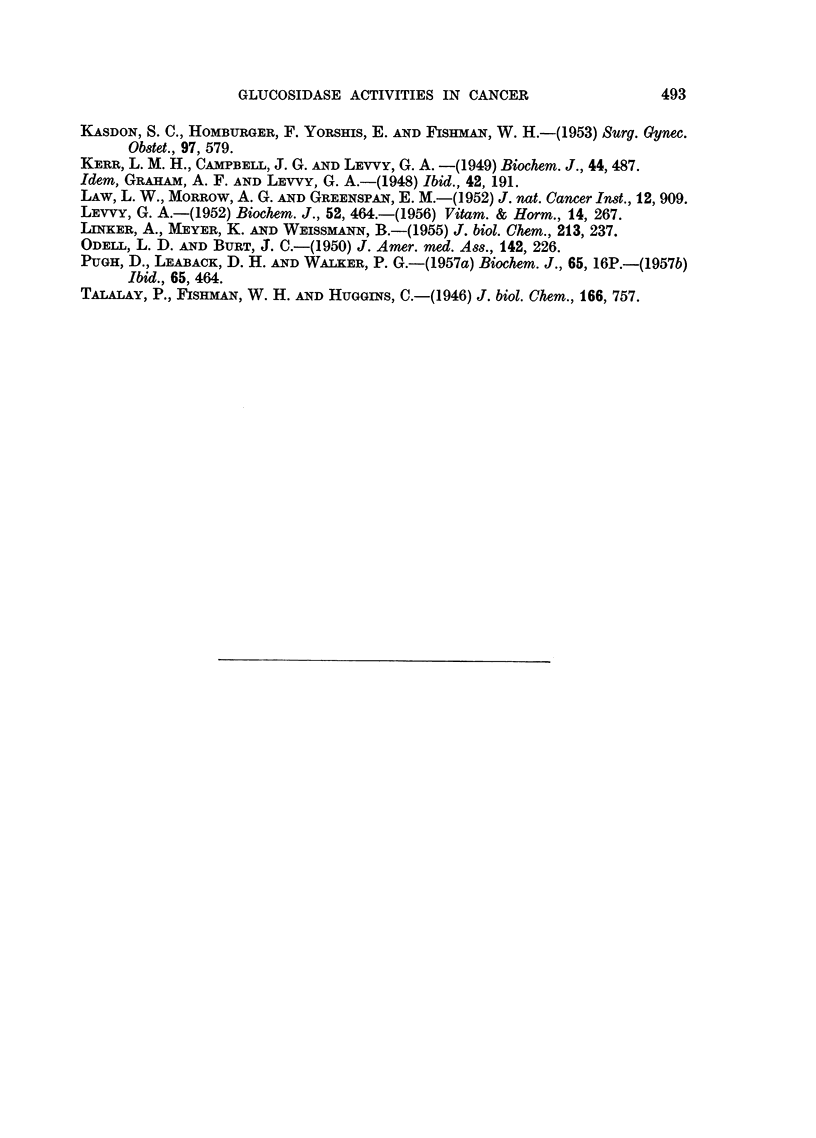


## References

[OCR_00568] CONCHIE J. (1954). Beta-Glucosidase from rumen liquor; preparation, assay and kinetics of action.. Biochem J.

[OCR_00570] CONCHIE J., FINDLAY J., LEVVY G. A. (1956). Alpha-mannosidase and other glycosidases in the tissues of the mouse and the rat, with special reference to the sex organs.. Nature.

[OCR_00579] FISHMAN W. H., FARMELANT M. H. (1953). Effects of androgens and estrogens on beta-glucuronidase in inbred mice.. Endocrinology.

[OCR_00584] KASDON S. C., HOMBURGER F., YORSHIS E., FISHMAN W. H. (1953). Beta-glucuronidase studies in women. VI. Premenopausal vaginal fluid values in relation to invasive cervical cancer.. Surg Gynecol Obstet.

[OCR_00588] Kerr L. M., Campbell J. G., Levvy G. A. (1949). beta-Glucuronidase as an index of growth in the uterus and other organs.. Biochem J.

[OCR_00589] Kerr L. M., Graham A. F., Levvy G. A. (1948). The use of phenol glucuronide in the assay of beta-glucuronidase.. Biochem J.

[OCR_00591] LAW L. W., MORROW A. G., GREENSPAN E. M. (1952). Inheritance of low liver glucuronidase activity in the mouse.. J Natl Cancer Inst.

[OCR_00592] LEVVY G. A. (1956). Glucuronide metabolism, with special reference to the steroid hormones.. Vitam Horm.

[OCR_00593] LINKER A., MEYER K., WEISSMANN B. (1955). Enzumatic formation of monosaccharides from hyaluronate.. J Biol Chem.

[OCR_00594] ODELL L. D., BURT J. C. (1950). New diagnostic adjunct for uterine cancer.. J Am Med Assoc.

